# Radioactive Iodine for Thyrotoxicosis in Childhood and Adolescence: Treatment and Outcomes

**DOI:** 10.4274/Jcrpe.951

**Published:** 2013-05-30

**Authors:** Sirianong Namwongprom, Kevalee Unachak, Prapai Dejkhamron, Supoj Ua-apisitwong, Molrudee Ekmahachai

**Affiliations:** 1 Chiang Mai University, Faculty of Medicine, Department of Radiology, Chiang Mai, Thailand; 2 Chiang Mai University Faculty of Medicine, Department of Pediatrics, Chiang Mai, Thailand

**Keywords:** Radioiodine treatment, thyrotoxicosis, children, adolescence, outcome

## Abstract

**Objective:** The aim of the present study was to evaluate the outcome of radioiodine treatment in thyrotoxicosis in childhood and adolescence.

**Methods:** This was a retrospective study of 27 patients (ages 7.2- 19.8 years) with a diagnosis of thyrotoxicosis who received iodine-131 (I-131) treatment from January 2007 to December 2011 in the Nuclear Medicine Division, Department of Radiology, Faculty of Medicine, Chiang Mai University. Gender, duration of antithyroid drug (ATD) treatment, 24-hour I-131 uptake, thyroid weight, total dose and number of treatments with I-131, and thyroid status at 6 months after treatment were recorded.

**Results:** The outcomes of 27 patients (85.2% female, 14.8% male) treated with radioactive iodine were analyzed to assess the effectiveness of therapy as related to dose and gland size. All children and adolescents received 150 µCi of I-131/g of thyroid tissue (n=27). Six 6 months after treatment, 44.5% of the patients were hyperthyroid, 14.8% were euthyroid, and 40.7% were hypothyroid. Of the 12 cases with hyperthyroidism, 2 cases needed a second dose of I-131 treatment, and they finally reached a hypothyroid state. The patients were classified into 2 groups according to treatment success (euthyroid and hypothyroid) and treatment failure (hyperthyroid). There were no significant differences in age, gender, duration of ATD treatment, 2- and 24-hour I-131 uptake, thyroid weight, and total I-131 dose between these two groups.

**Conclusions:** Radioiodine treatment is safe and effective for thyrotoxicosis in childhood and adolescence. It is suitable as a good second-line therapy for patients with severe complications, those who show poor compliance, and those who fail to respond to ATD treatment. .

**Conflict of interest:**None declared.

## INTRODUCTION

Thyrotoxicosis is rare in children and adolescents, affecting only 0.02% of all children (1). Graves’ disease (GD) is the most common cause of thyrotoxicosis in patients younger than 18 years of age and accounts for 10-15% of all paediatric thyroid diseases (2). GD is rare under the age of 5 years; it has a peak incidence at age 10-15 years and more commonly affects female patients (2,3). The management of thyrotoxicosis in children and adolescents remains controversial. Current therapeutic methods of thyrotoxicosis include treatment with antithyroid drugs (ATD), thyroidectomy, and radioactive iodine (RAI) therapy. Most paediatric endocrinologist prefer ATD medication as the first-line therapy. Definitive therapy (thyroidectomy or RAI treatment) is usually considered in cases of relapse, ATD toxicity, or lack of compliance.

Medical treatment by ATD is generally associated with a high relapse rate, risk of side effects including hepatic failure and bone marrow suppression, and low compliance associated with prolonged ATD therapy. Several studies reported that after ATD medications are discontinued, 35-60% of patients may experience relapse (3). Therefore, definitive therapy is favored as the first-line treatment in several countries. Thyroidectomy is associated with very high cure rates and a small risk of hypoparathyroidism and recurrent laryngeal nerve damage. Disadvantages of this method are surgical complications and hospitalization cost.

Due to its very high cure rate (exceeding 95%), there is now an increasing tendency to advocate RAI treatment as the first therapeutic modality in children and adolescents (4). When RAI is used at appropriate doses, most patients can be successfully treated with a single oral dose and very rare complications (4). The treatment effect is due to radioiodine-mediated destruction and also a particular effect on thyroid autoimmunity. In Chiang Mai University hospital, RAI treatment for paediatric hyperthyroidism was first introduced in 2007. The goal of this present study was to evaluate the outcome of RAI treatment and thus demonstrates the role of radioiodine in the treatment of hyperthyroidism in children and adolescents. 

## METHODS

Between January 2007 and December 2011, all children and adolescent patients who received iodine-131 (I-131) treatment for hyperthyroidism at the Nuclear Medicine Division, Department of Radiology, Faculty of Medicine, Chiang Mai University, were included in this retrospective study. All patients were diagnosed by paediatric endocrinologists on the basis of their clinical and laboratory findings. Gender, 2- and 24-hour I-131 uptake, thyroid weight, the total dose and number of treatments with I-131, and disease status at 6 months after treatment were recorded. The primary indication for I-131 treatment among all patients was failure of the medical treatment (92.6%). Two (7.4%) patients were referred to RAI treatment after experiencing adverse reactions to medical therapy. The thyroid I-131 uptake value was measured at 2 and 24 hours after an oral tracer dose (approximately 20 mCi) of I-131 through a I-131 uptake instrument (Captus 2000, Capintec Inc, USA). Thyroid volume (cm3) was calculated by ultrasound. All patients were treated with a fixed dose (150 µCi/g) of I-131. The actual given doses were then calculated depending on the thyroid weight of each patient. All patients were assessed for treatment outcome at 6 months after I-131 treatment. The patients were classified into 2 groups according to treatment success (euthyroid and hypothyroid) and treatment failure (hyperthyroid). The rate of treatment success was calculated. A Fisher exact test was used to test independence between binary variables. All data were analyzed and compared by using a STATA program version 11.0.

## RESULTS

During the study period, 27 patients (85.2% female, 14.8% male) with a mean age of 14.6 years (range, 7.2-19.8 years) received I-131 treatment for hyperthyroidism at our institution. All patients had been treated with either propylthiouracil or methimazole before receiving RAI. The outcomes of 27 patients treated with RAI were analyzed to assess the effectiveness of therapy as related to dose and gland size. This analysis showed that at 6 months after treatment, 44.5% of the patients were hyperthyroid, 14.8% were euthyroid, and 40.7% were hypothyroid. Of the 12 cases with hyperthyroidism, 10 were effectively controlled with ATD treatment. Two cases required a second dose of I-131 given within 6 months of the first RAI treatment to reach a hypothyroid state. There were no significant differences in age, gender, 2- and 24-hour I-131 uptake values, thyroid volume, and total I-131 dose between the treatment success and treatment failure groups (p<0.05) as shown in Table 1.

## DISCUSSION

Treatment of hyperthyroidism with RAI was introduced more than 60 years ago and was previously limited to adult patients. However, this treatment has lately become more popular in many countries for treating hyperthyroidism in children and adolescents (4,5,6,7). The goal of RAI treatment in hyperthyroidism is to achieve the hypothyroid state. To date, there is no consensus guideline on RAI treatment for hyperthyroidism in children. The dose used for treatment in children and adolescents varies among investigators (4,5,8,9,10,11,12,13). RAI dose is typically calculated based on thyroid gland volume and I-131 uptake. Some factors, including thyroid gland size and I-131 delivered dose to thyroid tissue, have been reported to influence treatment outcome (9,14). Rivkees et al (9) in 2003 reported hypothyroidism rates of 50%, 70%, and 95% obtained with I-131 doses of 100 Gy (110 µCi/g), 200 Gy (220 µCi/g), and 300 Gy (330 µCi/g), respectively, used in the treatment. They also recommended a treatment dose of 300 µCi/g to insure the hypothyroid state (9). Some investigators recommended a single fixed dose of I-131 for all patients (10,11,15). The recent management guidelines of the American Thyroid Association and the American Association of Clinical Endocrinologist published in 2011 recommend a treatment dose of >150 µCi/g of thyroid tissue to achieve hypothyroidism (16). In the present study, calculated doses of 150 µCi/g were prescribed for all patients.

Several studies have reported a very high remission rate (>95%) with doses >150 µCi/g (4,8,17). In the present study, we observed that 55.6% of our patients achieved the euthyroid and hypothyroid state after a single dose, which is a much lower rate than reported by other studies. Male sex, high free thyroxine at diagnosis, a palpable goiter, use of ATD, time elapsed before RAI treatment, and pre-existing ophthalmopathy have been reported as the predictors of poor treatment outcome (13,18). The results from this study showed no significant differences in age, gender, 2- and 24-hour I-131 uptake, thyroid volume, and total I-131 dose between the treatment success and treatment failure groups. None of the factors investigated were significantly related to outcome of the RAI treatment. All patients in this study have been previously treated with ATD before RAI treatment, which might have affected the low treatment outcome. Patients who do not respond well to medical treatment are more likely to have severe disease and also show a relative resistance to I-131 (13,19). The use of higher dose of RAI (200-300 µCi/g) might be suitable for this clinical situation.

Although properly administered RAI treatment is effective in children and adolescents who fail to respond to medical treatment, our data suggest that higher dose of I-131 may be suitable for this clinical setting. Further studies are needed to investigate the relationship between previous treatment with ATD and response to RAI treatment.

## Figures and Tables

**Table 1:**
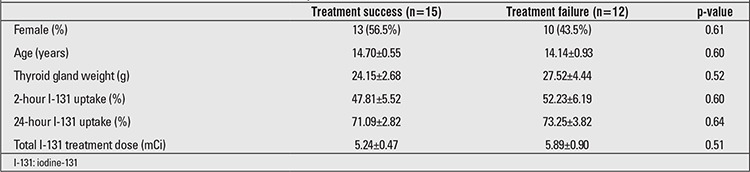
Characteristics of treatment success and failure subjects
